# Magnetic controlled capsule endoscope (MCCE)‘s diagnostic performance for *H. pylori* infection status based on the Kyoto classification of gastritis

**DOI:** 10.1186/s12876-022-02589-z

**Published:** 2022-12-06

**Authors:** Sun Xi, Liu Jing, Wu Lili, Li Tingting, Li Jun, Wang Ming, Wang Zhiqiang, Jin Peng

**Affiliations:** 1 Chinese PLA General Hospital, Department of Gastroenterology and Hepatology, The Second Medical Center and National Clinical Research Center for Geriatric Diseases, Beijing, China; 2 Department of Gastroenterology, The Seventh Medical Center of Chinese PLA General Hospital, Beijing, China

**Keywords:** Magnetic controlled capsule endoscope, *H. pylori*, Kyoto classification of gastritis, Gastric cancer, Early detection

## Abstract

**Background:**

Previous studies have shown that the Kyoto classification of gastritis can accurately predict *H. pylori* infection status on conventional gastroscopy. The aim of this study was to test whether the Kyoto classification of gastritis applies well to magnetic controlled capsule endoscopy (MCCE).

**Methods:**

We consecutively recruited 227 participants who underwent both MCCE and urea breath tests (UBTs). Two physicians who were blinded to the UBT results independently made the diagnosis of *H. pylori* infection status according to 10 findings listed in the Kyoto classification of gastritis after reviewing MCCE images. We also developed 2 predictive models to assess *H. pylori* infection status by combining these 10 findings.

**Results:**

The MCCE’s overall diagnostic accuracy for *H. pylori* infection status was 80.2%. The sensitivity, specificity and diagnostic odds ratio (DOR) for current infection were 89.4%, 90.1% and 77.1, respectively. Major specific findings were mucosal swelling and spotty redness for current infection, regular arrangement of collecting venules (RAC), streak redness, fundic gland polyp (FGP) for noninfection, and map-like redness for past-infection. In the two prediction models, the area under the curve (AUC) values for predicting noninfection and current infection were 84.7 and 84.9, respectively.

**Conclusions:**

The Kyoto classification of gastritis applied well to MCCE. *H. pylori* infection status could be accurately assessed on MCCE according to the Kyoto classification of gastritis.

**Supplementary Information:**

The online version contains supplementary material available at 10.1186/s12876-022-02589-z.

## Introduction

Gastric cancer (GC) now ranks as the world’s third leading cause of cancer-related death [1, 2]. As estimated by the Global Cancer Observatory (GCO), approximately 950,000 GCs are newly diagnosed every year, and the majority of these newly diagnosed GCs are reported from East Asian countries such as Japan, Korea and China [1, 3]. Early detection by esophagogastroduodenoscopy (EGD) can effectively reduce GC’s mortality rate. However, EGD is an invasive procedure that may raise concerns for patient discomfort and procedure-related adverse events (AEs), thus lowering patient compliance [3, 4].

Technological advances have led to the development of magnetic controlled capsule endoscopy (MCCE), a novel noninvasive device that can inspect the gastric mucosa in a contactless fashion [5–8]. In addition, MCCE does not require sedation, making it both a safer and a more comfortable screening modality than EGD. Moreover, recently published studies showed that MCCE’s diagnostic accuracy was comparable with that of conventional EGD [5, 6]. In recent years, MCCE has been developing rapidly and continues to gain popularity in China, where the prevalence of GC is the highest in the world [2, 9].

Despite these advantages, whether MCCE is sufficient to replace conventional EGD as a screening tool for GC has not been fully evaluated. One important issue that remains to be solved is the diagnosis of the *Helicobacter pylori* (*H. pylori*) infection status, as the risk of GC development is largely determined by one’s exposure to *H. pylori* [3, 9]. However, the diagnosis of *H. pylori* infection status on EGD is a challenging task even for experienced endoscopists.

In 2014, the Kyoto classification of gastritis was developed to facilitate the diagnosis of *H. pylori* infection status and better stratify the risk of GC using EGD. Most recent publications demonstrated that the Kyoto classification of gastritis was convenient and reliable in the three categorical diagnoses of *H. pylori* infection status [10, 11]. To date, however, whether the Kyoto classification of gastritis can be applied to MCCE remains unknown. Therefore, we conducted this study to validate whether the Kyoto classification of gastritis can be applied to MCCE and if *H. pylori* infection status could be accurately assessed on MCCE.

## Methods

### Study design

The diagnostic performance of MCCE in determining *H. pylori* infection status based on Kyoto classification was evaluated. We prospectively recruited individuals who came to our institute for a health check. These individuals either had mild epigastric symptoms or were totally asymptomatic. Participants were consecutively recruited from May 1 to December 31, 2019. The inclusion criteria were as follows: > 18 years old; scheduled for MCCE screening; and had urea breath test (UBT) results. Exclusion criteria were as follows: a history of gastric surgery; prior or current diagnosis of advanced GC; recent use of a proton pump inhibitor (PPI), histamine blocker, antibiotics, or bismuth; and suboptimal image quality.

A urea [13C] breath test (UBT) diagnostic kit (Beijing Huabo Medical Technology Co., Ltd.) was used for UBT, and all included participants were asked to fast overnight the day before UBT. The UBT was performed within 2 days before or after MCCE, and the results were regarded as the gold standard for *H. pylori* infection status. Current infection was considered if the UBT result was > 4 µmol/L, irrespective of *H. pylori* eradication history. Noninfection was considered if the UBT result was < 4 µmol/L. Past infection was considered when participants had a negative UBT result and clearly stated a history of successful *H. pylori* eradication more than 6 months before undergoing MCCE and UBT.

This study was conducted in accordance with the Helsinki Declaration and was approved by the ethics committee of PLA (People’s Liberation Army) General Hospital. All participants provided written informed consent.

### MCCE procedure

The MCCE used in this hospital was developed by Ankon Technologies Co., Ltd. (Shanghai, Wuhan, China). The participants were asked to fast overnight. Before swallowing the capsule, 2 L of water and simethicone were ingested to ensure a clear vision of the gastric mucosa. The examinations were conducted by an experienced technician (WM) according to the protocol described previously [12, 13].

### The diagnostic algorithm

**Fig. 1:**
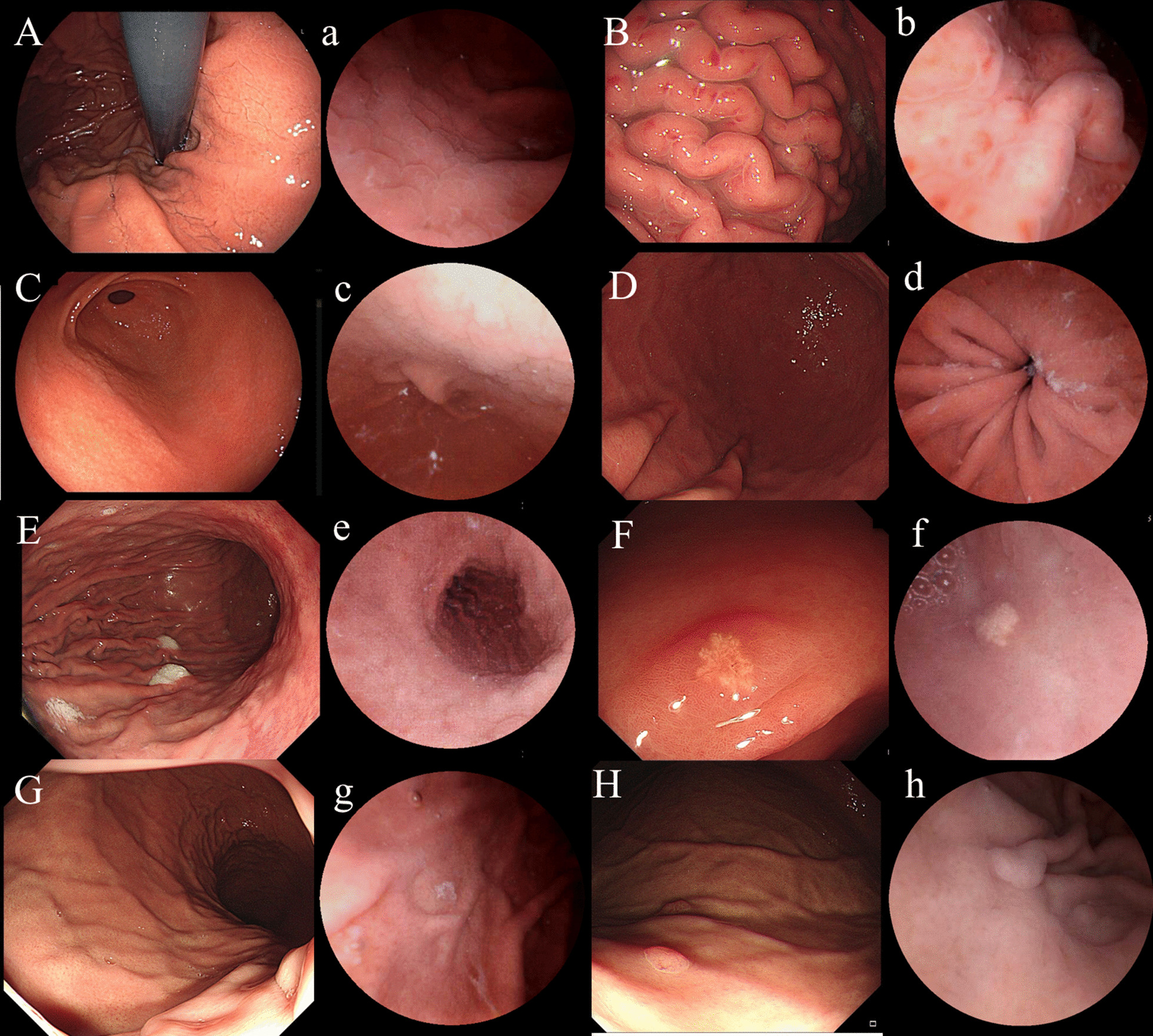
MCCE’s Kyoto classification of gastritis. (**A**–**H**, EGD images; **a**–**h** MCCE images). **A**, **a** mucosal swelling; **B**, **b** enlarged folds with spotty redness; **C**, **c** nodularity; **D**, **d** diffusive redness; **E**, **e** map-like redness; **F**, **f** xanthoma; **G**, **g** RAC; **H**, **h** FGP

According to relevant studies, we selected the following 10 findings listed in the Kyoto classification of gastritis that were closely related to *H. pylori* infection status: regular arrangement of collecting venules (RAC), fundic gland polyp (FGP), streak-like redness, xanthoma, map-like redness, spotty redness, diffuse redness, enlarged fold, mucosal swelling and nodularity [10, 11, 14]. The definition of MCCE’s Kyoto classification of gastritis is shown in Fig. 1. After reviewing MCCE’s real-time videos and still images, the diagnosis of *H. pylori* infection was made independently by an expert physician who had over 1,000 cases of capsule endoscope experience and a nonexpert physician who had less than 200 cases in experience. Both reviewers were blinded to the UBT results and *H. pylori* eradication history, and interobserver disagreements were resolved by a referee who was a veteran endoscopist with over 1000 capsule endoscope experience.

The diagnostic criteria for *H. pylori* infection status were established in a group session in which both reviewers were well versed in the Kyoto classification of gastritis [10, 11, 14, 15]. When at least two of the following findings were observed: spotty redness, diffuse redness, enlarged fold, mucosal swelling and nodularity, the diagnosis of current infection was made. When RAC, fundic gland polyp (FGP) or streak-like redness were observed and findings indicating current infection were not found, the diagnosis of noninfection was made. When map-like redness was observed alone or in combination with RAC or FGP, the diagnosis of past infection was made; additionally, if no significant findings for current infection or noninfection were observed, the diagnosis of past infection could also be considered [10, 11].

Two prediction models were developed to assess *H. pylori* infection status by combining 10 findings. In Model 1, noninfection participants were selected out of all included participants, and in Model 2, current infection participants were selected from those who were unselected in Model 1. Therefore, in Model 1, noninfection participants were identified, while in Model 2, current infection participants were identified.

### Statistical analysis

In our previous pilot study, we estimated that the sensitivity/specificity was approximately 80%/80%, and the required sample size was 62 in case of a 10% error allowance. The prevalence of *H. pylori* infection in our institution was approximately 30%; in that case, the total sample size required was 205. The estimated sample size was finally set at 220, considering that the drop rate of study participants was 10%.

R software (https://www.r-project.org) was used for statistical analysis. Continuous data are expressed as the mean value plus range. Diagnostic accuracy was calculated to evaluate MCCE’s overall diagnostic performance for *H. pylori* infection. Sensitivity, specificity, positive predictive value (PPV), negative predictive value (NPV) and diagnostic odds ratio (DOR) were calculated. Diagnostic parameters were expressed as values plus 95% confidential intervals (CIs). For each of the two prediction models, multivariate logistic regression analysis and receiver operating characteristic (ROC) curves for 10 findings were performed. In ROC analysis, the area under the curve (AUC) values were calculated to demonstrate the overall diagnostic performance. Interobserver variability was assessed by calculating kappa values. When kappa values were < 0.20, 0.21–0.40, 0.41–0.60, 0.61–0.80 and > 0.80, poor, fair, moderate, good and excellent agreement were rated, respectively.

## Results

### Baseline characteristics

There were 239 participants initially enrolled. However, 12 participants were excluded from the study because 3 participants had gastric surgery, 5 GERD patients were on PPIs and 4 participants had poor gastric preparation. Finally, 227 participants were enrolled. Their average age was 50.9 years old, with a range of 18 to 82 years old. There were 124 males and 103 females, and the male/female ratio was 1.20.

Among the 227 participants, the final diagnosis of *H. pylori* infection was current infection in 85 (85/227, 37.4%) participants, noninfection in 99 (99/227, 43.6%) participants and past infection (eradicated) in 43 (43/227, 18.9%) participants. Other diagnoses made on MCCE were gastro-esophageal reflux disease (GERD) in 18 (18/227, 7.9%) participants, submucosal tumor (SMT) in 15 (15/227, 6.6%) participants, telangiectasia in 13 (13/227, 5.7%) participants and bile reflux in 25 (25/227, 11.0%) participants. Erosions, either the elevated or flat type, were found in 95 (95/227, 41.9%) participants (Table 1).

**Table 1:** Baseline characteristics

Number of individuals (n)	227
Male (n, %)	124,54.6%
Female (n, %)	103,45.4%
M/F ratio	1.2
Age (years)	50.9(18–82)
Final diagnosis of *H. pylori* infection (n, %)	
Current-infection	90,39.6%
Noninfection	101,44.5%
Past-infection	36,15.9%
Other diagnoses on MCCE (n, %)	
Telangiectasia	13,5.7%
GERD	18,7.9%
SMT	15,6.6%
Erosive gastritis	95,41.9%
Bile reflux gastritis	25,11.0%

### Diagnostic performance of MCCE for *H. pylori* infection evaluated by Kyoto classification of gastritis

**Fig. 2:**
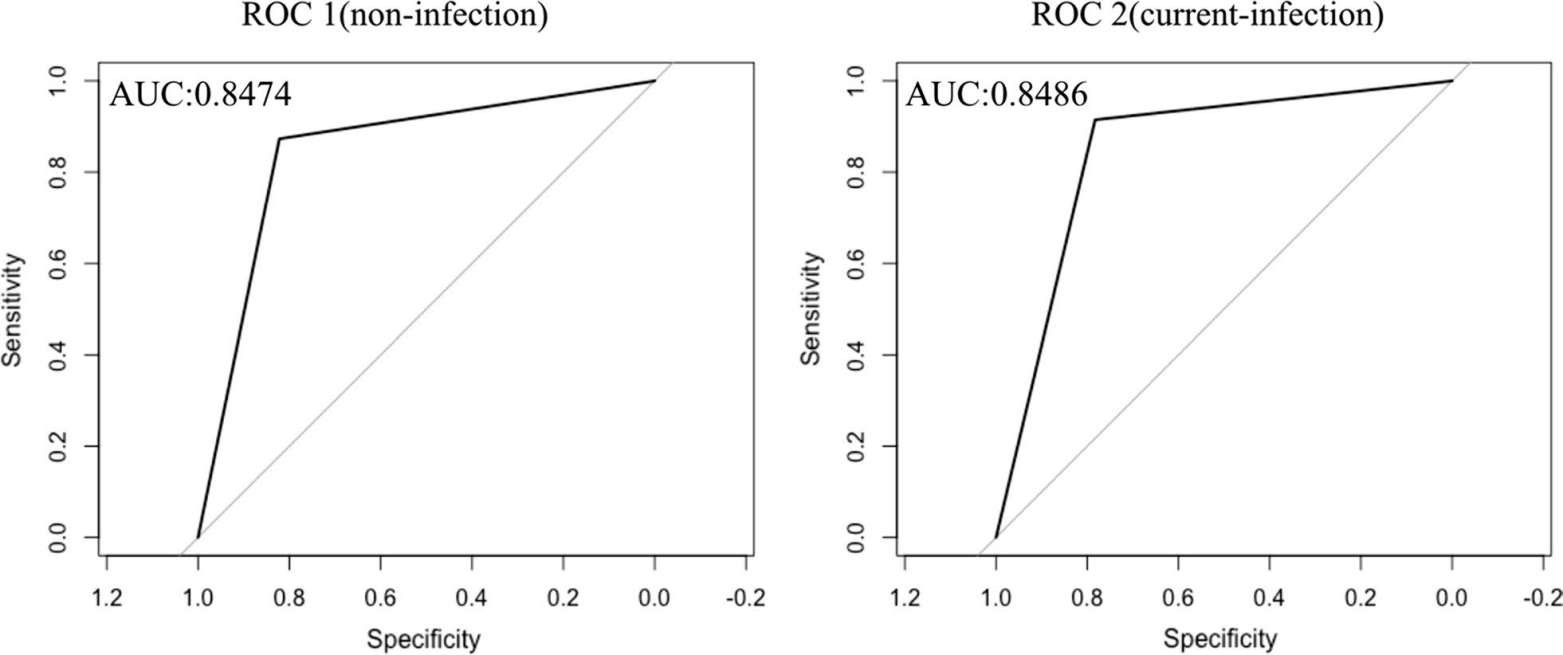
Receiver operating characteristic (ROC) curves for predicting noninfection (**A**) and current infection (**B**) based on 10 findings in the Kyoto classification of gastritis. AUC, area under the curve

Among the 227 participants who had undergone MCCE, 90 were diagnosed with current infection, 101 were diagnosed with noninfection and 36 were diagnosed with past infection. The overall diagnostic accuracy for *H. pylori* infection was 80.2% (182/227). The sensitivity, specificity, and PPV for current infection were 89.4%, 90.1%, and 9.07; for noninfection they were 83.8%, 85.9%, and 82.2%; and for past-infection they were 63.9%, 92.3%, and 53.5%, respectively (Table 2). The AUC for ROC1, which predicts noninfected individuals, was 84.7, and that of ROC2, which predicts current-infected individuals, was 84.9 (Fig. 2).

**Table 2:** Diagnostic performance of MCCE on *H. pylori* infection status

	Sensitivity (95% CI)	Specificity (95% CI)	PPV (95% CI)	NPV (95% CI)	DOR (95% CI)
Current infection n = 90	89.4% (81.2–95.3%)	90.1% (84.0–95.4)	84.4% (75.0–91.1%)	93.4% (87.6–96.9%)	77.2 (65.5–84.2)
Noninfection n = 101	83.8% (75.3–89.8%)	85.9% (78.6–91.0%)	82.2% (72.6–88.5%)	87.3% (79.8–93.2%)	30.7 (25.4–33.1)
Past-infection n = 36	53.5% (38.3–68.9%)	92.3% (87.7–95.5%)	63.9% (46.6–79.0%)	89.5% (83.7–92.7%)	15.1 (8.9–18.5)

### MCCE findings have high diagnostic value for a particular *H. pylori* infection status

Numerous endoscopic findings had high diagnostic value for indicating *H. pylori* infection status. Findings such as mucosal swelling (PPV 80.2%, DOR 25.6), diffusive redness (PPV 75.9%, DOR 9.2), spotty redness (PPV 79.2%, DOR 10.7), enlarged fold (PPV 85.7%, DOR 11.5) and nodularity (PPV 83.3%, DOR 8.3) were highly indicative of current infection. RAC (PPV 65.5%, DOR 7.7), streak-like redness (PPV 88.9%, DOR 12.1) and FGP (PPV 80.0%, DOR 6.2) were highly indicative of noninfection. For past infections, only map like redness (PPV66.7%, DOR14.0) had high diagnostic value (Additional file 1: Table S1, Additional file 2: Table S2, and Additional file 3: Table S3).

Mucosal swelling and spotty redness were simultaneously observed in 40 individuals. When this combination of findings was used as a diagnostic predictor for current infection, the PPV and DOR were 85.0% and 15.1, respectively. Among noninfected individuals, 19 had both FGP and RAC; the PPV and DOR of this combination for noninfection status were 89.5% and 13.1, respectively. For past infection, the combination of map like redness plus RAC was observed in 15 individuals, and this combination of findings yielded a PPV and DOR of 86.7% and 39.4, respectively (Additional file 1: Table S1, Additional file 2: Table S2, and Additional file 3: Table S3).

### Regression analysis

In predictive Model 1, RAC, FGP and streak-like redness were associated with noninfection. In predictive Model 2, mucosal swelling, spotty redness, diffusive redness, xanthoma and nodularity were associated with current infection, and these findings were inversely associated with noninfection in Model 1 (Table 3). Map-like redness had a negative regression coefficient in both Model 1 and Model 2 (Table 3).

**Table 3:** Regression coefficients for Model 1 (noninfection) and Model 2 (current infection)

Model 1 (noninfection)	Regression coefficient	Model 2 (current infection)	Regression coefficient
(Intercept)	0.59295	(Intercept)	0.72204
RAC	0.08841	RAC	− 0.32053
Map-like redness	− 0.34265	Map-like redness	− 0.43628
FGP	0.17032	FGP	− 0.43844
Mucosal swelling	− 0.38814	Mucosal swelling	0.13965
Spotty redness	− 0.20445	Spotty redness	0.22071
Diffuse redness	0.01439	Diffuse redness	0.11449
Xanthoma	− 0.04811	Xanthoma	0.0956
Streak-like redness	0.17907	Streak-like redness	− 0.03524
Enlarged fold	− 0.09648	Enlarged fold	0.35527
Nodularity	− 0.20485	Nodularity	0.25348

### Interobserver variability

Regarding the diagnosis of *H. pylori* infection status, the overall agreement was excellent, with a kappa value of 0.86. The kappa values for current infection and noninfection were 0.91 and 0.82, respectively, whereas the kappa value for past infection was relatively lower at 0.73.

Most of the 10 findings observed on MCCE had high kappa values and were rated as excellent or good agreement, except diffusive redness (Kappa value: 0.54), which was rated as moderate agreement (Additional file 4: Table S4).

## Discussion

One recently published Chinese study concluded that MCCE could detect GCs in a large population, but its role as a first-line screening tool for GC remains to be further validated [12]. Because the risk of GC is closely related to *H. pylori* infection status, MCCE’s diagnostic accuracy for *H. pylori* infection status is of critical importance in risk stratification. Moreover, the morphological features of early GC or high-grade precancerous lesions also differ according to different *H. pylori* infection statuses, which further established the rationale for our study.

Yoshii et al. demonstrated that the overall diagnostic accuracy of three *H. pylori* infection statuses was 82.9% on white light endoscopy by the Kyoto classification of gastritis [11]. In this study, we found that most of the key findings documented in the Kyoto classification of gastritis were recognizable on MCCE, *H. pylori* infection status could be accurately diagnosed via MCCE, and the overall diagnostic accuracy was 80.2%, comparable with EGD. Previous studies demonstrated that MCCE could detect various types of gastric lesions, including erosions, polyps, ulcers, and even superficial early gastric cancers [5, 9, 12, 16]. In our study, we found that the Kyoto classification of gastritis generally applied well to MCCE in the diagnosis of *H. pylori* infection status.

In the diagnosis of current infection status, the most reliable finding was mucosal swelling (sensitivity 76.5%, specificity 88.7%, PPV 80.2%), whereas in other recently published EGD studies, that diagnosis was established mainly based on observation of diffusive redness. This difference, we speculate, might have been the reason why MCCE had a higher DOR for current infection compared with conventional EGD (77.2 vs. 21.7) [10, 16, 17].

MCCE can reliably diagnose noninfection status, with a sensitivity, specificity and PPV of 83.8%, 85.0% and 82.2%, respectively. This diagnosis is mainly based on observation of RAC; although FGP and streak redness were also of high specificity and PPV, these two findings were relatively uncommon. However, MCCE’s DOR for noninfection status in our study was much lower than that of Yoshii’s EGD study (30.7 vs. 98.6), in which the authors made the diagnosis based on the same findings. The Kyoto gastritis classification defines RAC as microvascular networks observed in the lower part of the gastric corpus, mainly the lesser curve side [10, 14]. MCCE’s diagnostic performance on past-infection status was suboptimal in our study, largely due to the lack of specific findings. In addition, interobserver variability might also have played a role in its low diagnostic performance. A new discovery in our study was that the combination of RAC and map like redness could be used as a highly specific predictor for past infection, with a specificity, PPV and DOR of 98.9%, 86.7% and 39.4, respectively. This combination of findings is especially helpful for determining past infection status when there is diagnostic ambiguity.

Our study had several strengths. First, this was a prospective study in which the reviewers were blinded to the final results, and we used UBT results as the gold standard for the diagnosis of *H. pylori* infection, making the results reliable and robust. Second, we have found several combinations of findings with a high diagnostic value, which is useful when the diagnosis was uncertain based on observation of a single finding. Third, we performed regression analyses in which the diagnostic performance of MCCE was assessed by combining 10 findings in the Kyoto classification of gastritis. Fourth, we had an expert and a nonexpert review of MCCE images and resolved interobserver disagreement by a referee, making our results reproducible in future studies.

Our study had several limitations. First, although all participants were prospectively recruited, approximately half of the included participants were *H. pylori* noninfected (44.5%), while the proportion of past-infection participants was particularly low (15.9%); thus, according to STARD (Standards for reporting of diagnostic accuracy studies), selective bias was inevitable [18]. Second, according to the Kyoto classification of gastritis, sticky mucus and hyperplastic polyps are also key findings for *H. pylori* infection, but these findings were not included in our study, nor could we rate the degrees of atrophy and intestinal metaplasia on MCCE, so the scoring system of Kyoto classification of gastritis described in previous studies [19–22] was not available in this study. Therefore, the Kyoto classification of gastritis used in this study was actually a modified version [10–12, 14, 23]. Third, spontaneous eradication of *H. pylori* might have occurred in a small portion of the study participants, which could have impacted the evaluation of the diagnostic accuracy, which might have been underestimated in our study [10, 24].

In future studies, more specific findings for past infection are warranted because using map-like redness as the predictor does not appear to have sufficient diagnostic power. Additionally, in recent years, the introduction of artificial intelligence (AI) has improved the diagnostic accuracy of GI neoplasms as well as EGD’s diagnostic accuracy on *H. pylori* infection status [23, 25]. Hopefully, our results could help establish MCCE’s AI diagnosis of *H. pylori* infection status, thus improving GC’s early detection in a more reliable way [25, 26]. Moreover, efforts to establish scoring models for atrophy and intestinal metaplasia on MCCE are needed, which may help us better stratify GC risks via MCCE [19–21].

## Conclusions

The Kyoto classification of gastritis applied well to MCCE. *H. pylori* infection status could be accurately assessed on MCCE according to the Kyoto classification of gastritis.

## Supplementary Information

**Additional file 1.** Diagnostic value of significant endoscopic findings for current-infection.

**Additional file 2.** Diagnostic value of significant endoscopic findings for noninfection.

**Additional file 3.** Diagnostic value of significant endoscopic findings for past-infection.

**Additional file 4.** Inter-Observer agreement on 10 MCCE findings.

## Data Availability

The data that support the findings of this study are available from the corresponding author upon reasonable request.

## Abbreviations

AI: Artificial intelligence
CI: Confidence interval
DOR: Diagnostic odds ratio
EGD: Esophagogastroduodenoscopy
FGP: Fundic gland polyp
GC: Gastric cancer
GERD: Gastroesophageal reflux disease
GCO: Global Cancer Observatory
*H. pylori*: *Helicobacter pylori*
MCCE: Magnetic controlled capsule endoscopy
NPV: Negative predictive value
PPI: Proton pump inhibitor
PPV: Positive predictive value
RAC: Regular arrangement of collecting venules
STARD: Standards for reporting of diagnostic accuracy studies
SMT: Submucosal tumor
UBT: Urea breath test
