# Supplementary material for: Magnetic controlled capsule endoscope (MCCE)‘s diagnostic performance for H. pylori infection status based on the Kyoto classification of gastritis
Source: BMC Gastroenterol. 2022 Dec 6;22:502. doi: 10.1186/s12876-022-02589-z (PMC9724339; doi:10.1186/s12876-022-02589-z)
Supplement: Supplementary file 1 — Additional file 1. Diagnostic value of significant endoscopic findings for current-infection. [file 12876_2022_2589_MOESM1_ESM.docx]

**Supplementary table 1. Diagnostic value of significant endoscopic findings for current-infection**

|  | **sensitivity**  (95%CI) | **specificity**  (95%CI) | **PPV**  (95%CI) | **NPV**  (95%CI) | **DOR**  (95%CI) |
| --- | --- | --- | --- | --- | --- |
| mucosal  swelling | 76.5%  (65.6%-85.1%) | 88.7%  (82.1%-93.1%) | 80.2%  (69.9%-88.3%) | 86.3%  (80.0%-91.4%) | 25.6  (19.3-31.5) |
| diffusive  redness | 48.2%  (37.3%-58.9%) | 90.8%  (84.5%-95.4%) | 75.9%  (62.1%-87.3%) | 74.6%  (66.7%-81.0%) | 9.2  (4.5-14.2) |
| spotty  redness | 44.7%  (34.4%-56.2%) | 93.0%  (86.7%-97.1%) | 79.2%  (65.2%-90.0%) | 73.7%  (67.1%-80.2%) | 10.7  (7.6-16.1) |
| enlarged  fold | 14.1%  (7.8%-23.1%) | 98.6%  (94.5%-99.5%) | 85.7%  (56.9%-98.1%) | 65.7%  (58.9%-71.5%) | 11.5  (6.4-18.6) |
| nodularity | 5.8%  (2.3%-13.4%) | 99.3%  (96.1%-99.7%) | 83.3%  (35.6%-97.6%) | 63.8%  (57.0%-70.3%) | 8.8  (3.8-14.4) |
| xanthoma | 12.9%  (7.4%-21.5%) | 95.1%  (90.3%-97.8%) | 61.1%  (35.6%-83.2%) | 94.6%  (90.1%-98.2%) | 2.5  (0.8-4.4) |
| spotty redness  mucosal swelling  * | 40.0%  (29.9%-51.2%) | 95.8%  (91.1%-98.0%) | 85.0%  (70.3%-93.8%) | 77.7%  (71.5%-82.7%) | 15.1  (11.4-22.6) |

*spotty redness plus mucosal swelling
