# Supplementary material for: Magnetic controlled capsule endoscope (MCCE)‘s diagnostic performance for H. pylori infection status based on the Kyoto classification of gastritis
Source: BMC Gastroenterol. 2022 Dec 6;22:502. doi: 10.1186/s12876-022-02589-z (PMC9724339; doi:10.1186/s12876-022-02589-z)
Supplement: Supplementary file 2 — Additional file 2. Diagnostic value of significant endoscopic findings for noninfection. [file 12876_2022_2589_MOESM2_ESM.docx]

**Supplementary table 2. Diagnostic value of significant endoscopic findings for non-infection**

|  | **sensitivity**  (95%CI) | **specificity**  (95%CI) | **PPV**  (95%CI) | **NPV**  (95%CI) | **DOR**  (95%CI) |
| --- | --- | --- | --- | --- | --- |
| RAC | 76.8%  (67.2%-84.5%) | 68.8%  (60.0%-77.4%) | 65.5%  (56.1%-74.4%) | 79.3%  (71.0%-85.6%) | 7.7  (5.5-15.9) |
| streak  redness | 16.2%  (10.1%-25.4%) | 98.4%  (93.5%-100.0%) | 88.9%  (64.5%-98.9%) | 60.3%  (53.0%-67.2%) | 12.1  (8.8-16.8) |
| FGP | 20.2%  (13.1%-28.9%) | 96.1%  (90.5%-99.1%) | 80.0%  (58.9%-93.3%) | 60.9%  (54.4%-67.8%) | 6.2  (3.3-8.5) |
| RAC  FGP * | 16.7%  (10.3%-25.6%) | 98.4%  (94.4%-100.0%) | 89.5%  (67.1%-98.9%) | 60.6%  (54.0%-66.7%) | 13.1  (9.6-16.3) |

*****RAC plus FGP
