# Supplementary material for: Magnetic controlled capsule endoscope (MCCE)‘s diagnostic performance for H. pylori infection status based on the Kyoto classification of gastritis
Source: BMC Gastroenterol. 2022 Dec 6;22:502. doi: 10.1186/s12876-022-02589-z (PMC9724339; doi:10.1186/s12876-022-02589-z)
Supplement: Supplementary file 3 — Additional file 3. Diagnostic value of significant endoscopic findings for past-infection. [file 12876_2022_2589_MOESM3_ESM.docx]

**Supplementary table 3. Diagnostic value of significant endoscopic findings for past-infection**

|  | **sensitivity**  (95%CI) | **specificity**  (95%CI) | **PPV**  (95%CI) | **NPV**  (95%CI) | **DOR**  (95%CI) |
| --- | --- | --- | --- | --- | --- |
| RAC | 67.4%  (51.4%-81.1%) | 52.7%  (45.0%-60.2%) | 25.0%  (16.7%-34.1%) | 87.4%  (80.1%-92.6%) | 2.3  (0.8-4.2) |
| map-like  redness | 41.9%  (26.7%-58.2%) | 95.1%  (91.1%-97.8%) | 66.7%  (45.6%-83.3%) | 87.5%  (82.2%-91.9%) | 14.0  (10.5-19.2) |
| xanthoma | 9.3%  (2.9%-22..2%) | 92.3%  (88.0%-96.1%) | 22.2%  (5.6%-48.3%) | 81.3%  (74.5%-85.8%) | 1.2  (0.7-2.3) |
| map-like  redness  RAC* | 30.3%  (16.5%-45.8%) | 98.8%  (96.0%-99.8%) | 86.7%  (60.3%-97.8%) | 85.8%  (80.1%-89.8%) | 39.4  (25.6-53.4) |

*map-like redness plus RAC
