# Supplementary material for: Magnetic controlled capsule endoscope (MCCE)‘s diagnostic performance for H. pylori infection status based on the Kyoto classification of gastritis
Source: BMC Gastroenterol. 2022 Dec 6;22:502. doi: 10.1186/s12876-022-02589-z (PMC9724339; doi:10.1186/s12876-022-02589-z)
Supplement: Supplementary file 4 — Additional file 4. Inter-Observer agreement on 10 MCCE findings. [file 12876_2022_2589_MOESM4_ESM.docx]

**Supplementary table 4.Inter-Observer agreement on 10 MCCE findings.**

| **Findings on MCCE** | **Kappa value** |
| --- | --- |
| mucosal swelling | 0.82 |
| diffusive redness | 0.54 |
| spotty redness | 0.95 |
| enlarged fold | 0.88 |
| nodularity | 0.83 |
| atrophy | 0.75 |
| xanthoma | 0.87 |
| RAC | 0.84 |
| streaky redness | 0.86 |
| FGP | 0.98 |
| map redness | 0.77 |
